# Apoptotic Effect of Morphine, Imiquimod and Nalmefene on Promastigote, Infected and Uninfected Macrophages with Amastigote of *Leishmania Major* by Flow Cytometry

**Published:** 2018

**Authors:** Parisa Ebrahimisadr, Fatemeh Ghaffarifar, John Horton, Abdolhosein Dalimi, Zohreh Sharifi

**Affiliations:** a *Department of Parasitology, Faculty of Medical Sciences, Tarbiat Modares University, Tehran, Iran.*; b *Tropical Projects, Hitchin, United Kingdom. *; c *Blood Transfusion Research Center, High Institute for Research and Education in Transfusion Medicine, Tehran, Iran.*

**Keywords:** Morphine, Imiquimod, Nalmefene, Apoptosis, *Leishmania major*

## Abstract

The parasites of genus Leishmania are the causative agents of one of the most widespread and devastating diseases**.** According to follow-up data, these medications may provoke adverse drug reactions, drug resistance, relapse, as well as financial burden**.** The mechanism of action of opioid drugs is primarily exerted via transmembrane G-protein coupled receptors. One of the potent synthetic immunomodulator agents is imiquimod with low molecular weight and unknown mechanism of action. Monocyte and macrophage are the primary site of action for imiquimod. Nalmefene is a well-known opioid antagonist agent which simultaneously inhibits these receptors and augments intracellular pathogenicity, hence providing opportunities to investigate their function. The aim of present work was evaluating the effect of morphine, imiquimod and nalmephen on the *Leishmania major *and investigating cytotoxic effect this drug on the uninfected macrophage and infected macrophage for detected early apoptosis, necrosis and secondry apoptosis by flowcytometry method. In this study we used morphine, imiquimod, nalmefene, and Glucantime. We treated promastigotes, macrophages, and infected macrophages with above drugs, and the apoptosis evaluated by flow cytometry. The results showed that in all concentration of morphine more than 98% of promastigotes remained alive that it is deduced that morphine lacks any lethal effect on *L. major* after 24 h, whereas in groups treated with Glucantime alone or in combination with Nalmephene and Imiquimod, 84.13%, 88.96% and 86.72% of promastigotes were alive, respectively. The results of macrophage treatment with morphine, imiquimod, and nalmefene demonstrated that most necrosis has occurred in nalmefene group (6.54%).

## Introduction

The parasites of genus Leishmania (Family Trypanosomatidae) are the causative agents of one of the most widespread and devastating diseases, i.e. Leishmaniasis, which are transmitted by sandflies (1). The macrophages of each infected organ act as the main residence for Leishmania; depended on the infection site, leishmaniasis manifests as three types of complications, including cutaneous, mucocutaneous, and visceral (2-3). Cutaneous Leishmaniasis (CL) is prevalent in 98 nations, most of them in Afghanistan, Algeria, Brazil, Iran, and Syria (4-5).The outcomes of cutaneous form ranges from tiny, non-progressive nodules to ulcerative lesions. There are several, common chemical compounds which are used for treatment of CL, such as Glucantim, Pentostam, Allopurinol and Alluporinol riboside, Paramomycin, aromatic diamidines and polyene antibiotics (Amphotericin B). Although, according to follow-up data, these medications may provoke adverse drug reactions, drug resistance, relapse, secondary bacterial infections, as well as financial burden (6). The mechanism of action of opioid drugs are primarily exerted via transmembrane G-protein coupled receptors. Upon binding morphine to the extracellular domain of these receptors, signal transduction would occur toward the cytoplasm; subsequently, the ordinary functions of the immune cells, especially macrophages and lymphocytes, are changed owing to a series of molecular cascades (7-8).One of the potent synthetic immunomodulator agents is an imidazoquinoline compound, i.e. Imiquimod (1-(2-methylprophyl)-1H-imidazo (4,5c) quinoline- 4-amine) with low molecular weight and unknown mechanism of action. Monocyte and macrophage are the primary site of action for imiquimod. This topical compound would trigger monocytes and dendritic cells via Th1-biased immune responses (9-10). Imiquimod is used in many complications such as genitoanal warts, actinic keratoses, basal cell carcinoma, Kaposi’s sarcoma, and chronic hepatitis C. Moreover, in systemic administration, efficient and promising imiquimod function has been determined in some metastatic tumors consisting of melanomas, lung sarcomas, and mammary carcinomas (11). The important cells for imiquimod are monocytes/macrophages (12-14).

Although the mechanism of action of this drug is not well understood (15-18).Having remarkable affinity for triple opioid receptors, Nalmefene is a well-known opioid antagonist agent which simultaneously inhibits these receptors and augments intracellular pathogenicity, hence providing opportunities to investigate their function. (19)

Programmed cell death or apoptosis is a diversity of cellular morphological (blebbing, cell shrinkage, nuclear fragmentation, chromatin condensation, chromosomal DNA fragmentation) and biochemical (externalization of phosphatidyl serine in the plasma membrane, oxidative stress, release of proteins from mitochondria e.g., cytochrome c, endonuclease G, apoptosis –inducing factor ,activity of proteases e.g., caspases) changes which may occur physiologically or pathologically (20-21) .

The aim of present work was evaluating the effect of morphine, imiquimod, and nalmefen on the *Leishmania major *and investigating cytotoxic effect of this drug on the uninfected macrophage and infected macrophage for detected early apoptosis, necrosis, and secondry apoptosis by flowcytometry method.

## Experimental

*Etical statement*

This study was approved in Medical Ethics Committee on 1394/11/20, with issue number 52/D/8207.

*Parasite culture*

This study is an experimental study*. L.major* ((MRHO/IR/75/ER) was obtained from parasitology department of Tarbiat Modares University. Promastigotes were cultured in RPMI 1640 (Gibco,US) enriched with FBS 20% (Fetal Bovine Serum) (Gibco, US), 100 IU/mL penicillin and 100 µgr/mL streptocillin, then incubated in 24 °C.

*Drug preparation*

Drug preparations for Morphine, Imiquimod, Nalmefen, and Glucantime were as follow: 

Morphine sulfate (powder) was purchased from Temad, Iran. We used morphin with 0.1, 10 and 100 µg/mL concentrations.

Imiquimod was as dry powder, being purchased from InvitroGen-San Diego, USA. 1 mg of powder was dissolved in 1 mL of its specific solvent (available commercially).We used 0.01, 0.1and 1 µg/mL concentrations prepared with RPMI 1640. 

Nalmefen (selincro) was purchased from Selincro France. Nalmefen was used in 0.01, 1, and 10 µg/ml concentrations. At first, it was solved in DMSO and then diluted in RPMI 1640. 

Glucantime was purchased as a 300 mg/mL liquid solution from Sanofi-avetis France. Glucantime was used in 50 µg/mL concentration. 

*Macrophage culture:*

In this study, J774 A1 (Mouse CGBR-80052901, kindly offered by Professor Marcel Hommel) macrophages were used. At first, J774 was cultured in RPMI 1640 with FBS10%, 100 IU/mL penicillin, 100 µg/mL streptomycin, then incubated in 37 °C and 5% CO2 atmosphere. 

*Infected macrophage with leishmania major:*

J774 A1 macrophage cells were grown in RPMI 1640 plus 10% FBS, in a humidified atmosphere of 95% air and 5% CO2 at 37 °C. 1mL J774 A1 macrophage cells were seeded at a concentration of 1×10^5^cells/well in 12-well microplates (Nunc) and cultured for 24 h.

*Treatment uninfected macrophage, infected macrophage and promastigote of leishmania major with drugs*

*Uninfected macrophage*

Morphin with different concentrations (0.1, 10, 100µg/mL), imiquimod (0.01µg/mL), nalmefen (0.01µg/mL), Glucantime (50µg/mL), morphin (0.05µg/mL) + imiquimod (0.05µg/mL), morphin (5µg/mL) +nalmefen (5µg/mL), Glucantime (25µg/mL) + imiquimod (0.5µg/mL), Glucantime (25µg/mL) + nalmefen (0.5µg/mL) was added to macrophages. One well consist of 1mL J774 A1 macrophage Cells and 1mL RPMI 1640 plus 10% FBS (control group). After 24 h of incubation the macrophages were collected for flowcytometry assay 

*Infected macrophage*

J774 A1 macrophage Cells were seeded at a concentration of 1×10^5^cells/well in 12-well microplates (Nunc) cultured for 24 h. The cells were then infected *in-vitro* with promastigote of *L. major *in stationary phase at a ratio of 10:1. After 6 h of incubation, non-phagocytic parasites were removed by washing. Infected macrophages were further incubated in the presence of different drugs and concentrations as mentioned above or absence (negative control group).

*Promastigote of leishmania major*

10^6^ promastigote of *leishmania major* was cultured with different drugs and concentrations as mentioned above, then incubated in 24 °C. After 24 h the promastigote was collected for flowcytometry assay.

*Apopptic assay by Flowcytometry:*

The Annexin-V FLUOS Staining Kit (Bio-vision, USA) was used for the detection of apoptotic and necrotic cells. Flowcytometry analysis assessed to infected and uninfected macrophage cells and promastigotes. The cells were collected after 24 h incubation and centrifuged at 3000 rpm for 5 min, then supernatant was discharged, and 500μL binding buffer, 5μL annexin V and 5μL propidium iodide (PI) were added to the residue. The samples incubated at room temperature and dark situation for 5 min. Then, they were obtained by BD FACSCantoII flow cytometer (BD Biosciences, San Jose, CA) and were analysed by flowing software2.

## Results

Among three concentrations of morphine (0.1, 10 and 100 µg/mL) more than 98% of promastigotes remained alive. Regarding 99.06% survival rate in control group, it is deduced that morphine lacks any lethal effect on *L. major* after 24 h whereas in groups treated with Glucantime alone or in combination with nalmefen and imiquimod, 84.13%, 88.96% and 86.72% of promastigotes were alive, respectively. No difference was observed in terms of alive macrophages in various concentrations of morphine-treated macrophage group and the rate of insignificant apoptosis and necrosis in groups with various morphine concentrations (0.1, 10 and 100 µg/mL) were 8.11, 5.22, and 7.02, respectively.

**Figure.1:**
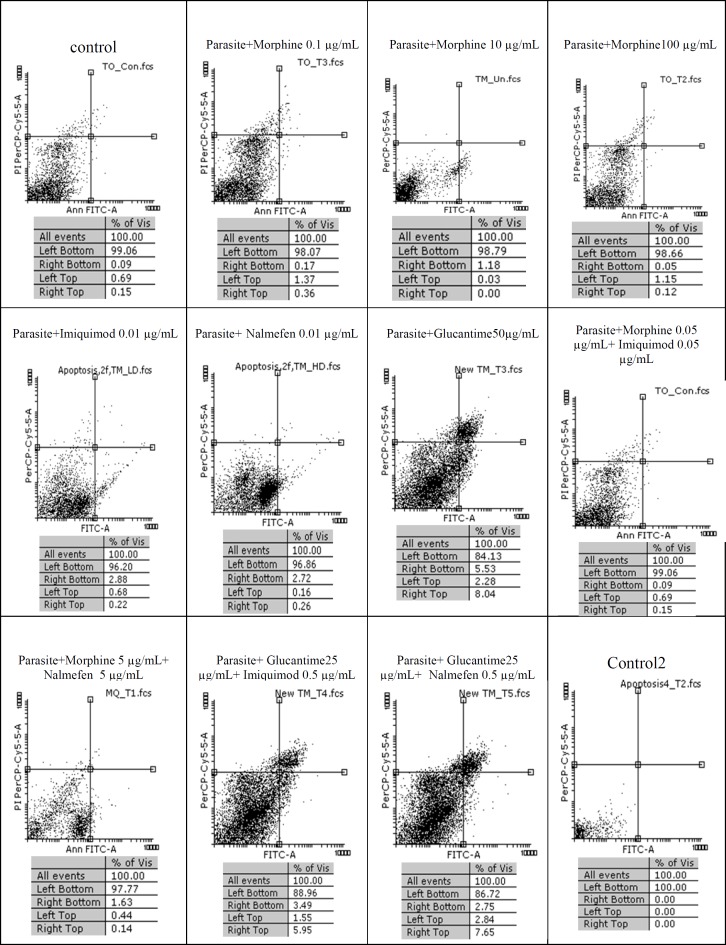
Flow cytometry results. Promastigotes staining with Annexin V and Propidium Iodide after treatment with different concentrations of morphine, imiquimod ,nalmefen and Glucantime after 24 h. Regions of quardrate show necrosis cells (Propidium Iodide positive) in left top, late apoptosis in Right top, Right bottom region belongs to apoptotic cells (annexin positive) and left bottom region belongs to live cells.

**Figure.2:**
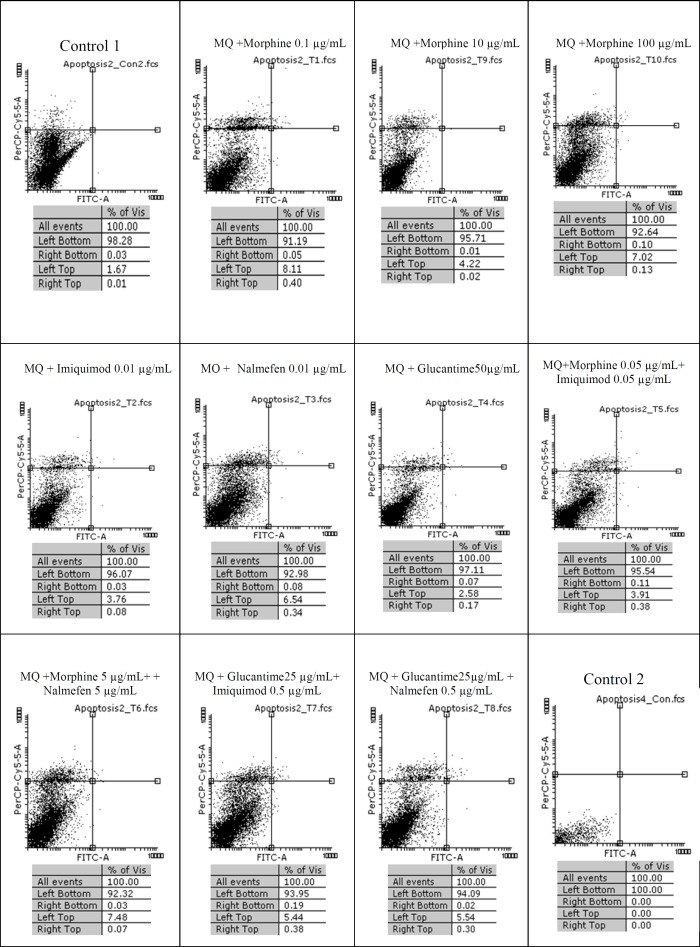
Flow cytometry results. Macrophage staining with Annexin V and Propidium Iodide after treatment with different concentrations of morphine, imiquimod, nalmefen and Glucantime after 24 h. Regions of quardrate show necrosis cells (Propidium Iodide positive) in left top,late apoptosis in Right top, Right bottom region belongs to apoptotic cells (annexin positive) and left bottom region belongs to live cells

**Figure.3:**
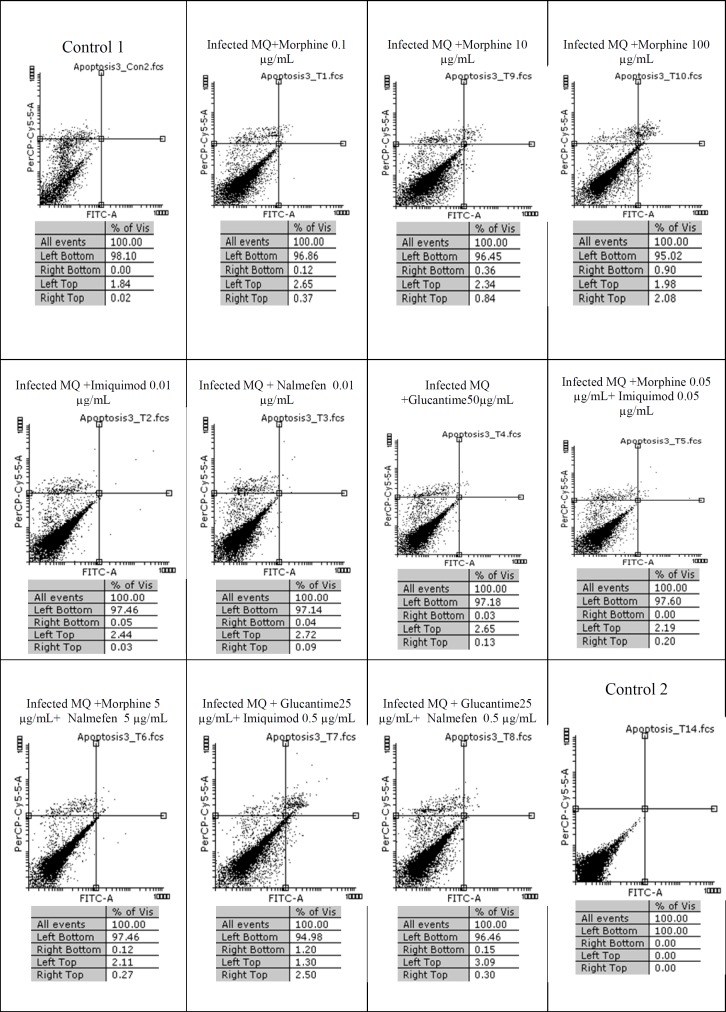
Flow cytometry results Infected macrophage staining with Annexin V and Propidium Iodide after treatment with different concentrations of morphine, imiquimod , nalmefen and Glucantime after 24 h. Regions of quardrate show necrosis cells (Propidium Iodide positive) in left top, late apoptosis in Right top, Right bottom region belongs to apoptotic cells (annexin positive) and left bottom region belongs to live cells.

The results of macrophage treatment with morphine, imiquimod, and nalmefen demonstrated that most necrosis has occurred in nalmefen group (6.54%). In *L. major*-infected macrophages treated with morphine, the most cytotoxicity was associated with morphine 0.1% concentration. Additionally, nalmefen group represented more lethal effects rather than imiquimod group. We added Control 1 groups annexin V and propidium iodide (PI) and Control 2 groups were without annexin V and propidium iodide (PI). Other information are illustrated in Figures 1, 2, and 3.

## Discussion

Cutaneous Leishmaniasis is a cosmopolitan disease with several forms of lesions. In this study, we evaluated the effects of morphine, imiquimod, and nalmefen on apoptosis of promastigotes and macrophages infected with *Leishmania major* amastigotes. The so-called metacaspases as analogues of caspase enzymes inflict apoptosis in the Leishmania parasites (22-24). In study about incidence of cutaneous leishmaniasis in Iran was calculated as 22 per 100000 population (25). Leishmaniasis caused by *Leishmania major* is endemic in northeast, south, and central areas in Iran (26-27 ).The annual cases of cutaneuse leishmaniasis in Iran is approximately 20,000–30,000 cases reported (28 ).

Macrophages are central to host’s innate and acquired immune system as phagocytize and secrete target-specific cytokines. Apoptosis is inhibited in infected macrophages by *Leishmania donovani* and *Leishmania major* through removing macrophage colony stimulating factor (M-CSF). Also, it has been demonstrated that caspase 3 activation is blocked by *Leishmania major* via impeding the release of cytochrome C from mitochondria (29-30). Hence, trigerring apoptosis is one of the successful mechanisms to wipe out intracellular infections (31-32).Previous studies have indicated the immunomodulatory impacts of opioids on different parasitic diseases. At least a biphasic role of *L. donovani* and *P. bergei *infection modulation was observed by morphine through macrophage-derived protective artillery (33-34).

An intricated, highly interlocked mesh of receptors and ligands between the immune and the neuroendocrine systems mediate the opioid immunomodulation pathways (35-36). One of the important sites of action for opioids is macrophage. The function of morphine is including the alteration of the burst phenomenon, as the principle protective response to infections, as well as the phagocytic acting of these cells. Nevertheless, morphine can enhance and stimulate the immune responses. The potency of morphine to perform its inhibitory actions is relied on the observed dose-dependent impacts of this drug, whereas the length of inhibition course is concentration-dependent. A stage of hyperactivity would occur in inhibited cells, which then turns into immunostimulation. Subsequently, the cells challenged with low concentrations, return quicker to the stimulatory phase, causing enhanced immune activities such as phagocytosis (37).Accordingly, the protective effects of morphine may be attributed to the macrophage-derived mechanisms. This fact has been proved by elimination of these protective effects using an optional macrophage killing agent, i.e. silica (38).

The results of our work showed that in uninfected macrophage groups or parasite-infected, little apoptosis was observed during treatment with several concentrations of morphine, imiquimod, and nalmefen. In control group of parasite-infected macrophages or uninfected macrophages no remarkable difference was considered in terms of apoptosis and necrosis. The rate of apoptosis was low in infected and uninfected macrophages treated with morphine, while the rate of necrosis was more prominent in uninfected macrophages. 

Also, necrosis rate was higher in uninfected macrophages treated with different morphine concentrations comparable to infected macrophages. The cytotoxicity effects of morphine, nalmefen, and imiquimod were lower than Glucantime. No significant effects were observed on promastigotes treated with morphine, nalmefen, and imiquimod, while Glucantime is induced early (5.53%) and late (8.04%) apoptosis. Furthermore, Glucantime combined to imiquimod and nalmefen have impelled apoptosis in promastigotes and apoptotic effects more remarkable in glucantime alone group in comparison to Glucantime with imiquimod and nalmefen Regarding to previous studies demonstrating positive effects of morphine for treatment of infectious diseases as well as data achieved during this research indicating lack of lethal effects of morphine on parasites and infected macrophages, it can be deduced that morphine acts by improving immune system. The effect of nalmefen has not yet been evaluated in infectious diseases. Based on the results of this study, morphine and imiquimod possess immunomodudlatory effect and could be used as a novel therapeutic procedure alone or combined to other drugs or they can enhance current treatment options.
